# (μ-1,2-Di-4-pyridylethyl­ene-κ^2^ *N*:*N*′)bis­[bis­(*N*,*N*-dimethyl­dithio­carbamato-κ^2^ *S*,*S*′)zinc(II)]

**DOI:** 10.1107/S1600536809044249

**Published:** 2009-10-31

**Authors:** Pavel Poplaukhin, Edward R. T. Tiekink

**Affiliations:** a Chemical Abstracts Service, 2540 Olentangy River Rd, Columbus, Ohio 43202, USA; b Department of Chemistry, University of Malaya, 50603 Kuala Lumpur, Malaysia

## Abstract

The dinuclear title compound, [Zn_2_(C_3_H_6_NS_2_)_4_(C_12_H_10_N_2_)], features two five-coordinate Zn atoms, one with an NS_4_ coordination geometry distorted towards a trigonal-bipyramidal arrangement, and the other distorted towards a square pyramid. In the crystal, mol­ecules are connected into supra­molecular zigzag chains *via* C—H⋯S contacts. Chains are connected *via* C—H⋯π interactions, consolidating the crystal packing.

## Related literature

For background to supra­molecular polymers of zinc 1,1-dithiol­ates, see: Lai *et al.* (2002 ▶); Chen *et al.* (2006 ▶); Benson *et al.* (2007 ▶). For a related structure and the synthesis, see: Lai & Tiekink (2003 ▶). For additional geometrical analysis, see: Addison *et al.* (1984 ▶).
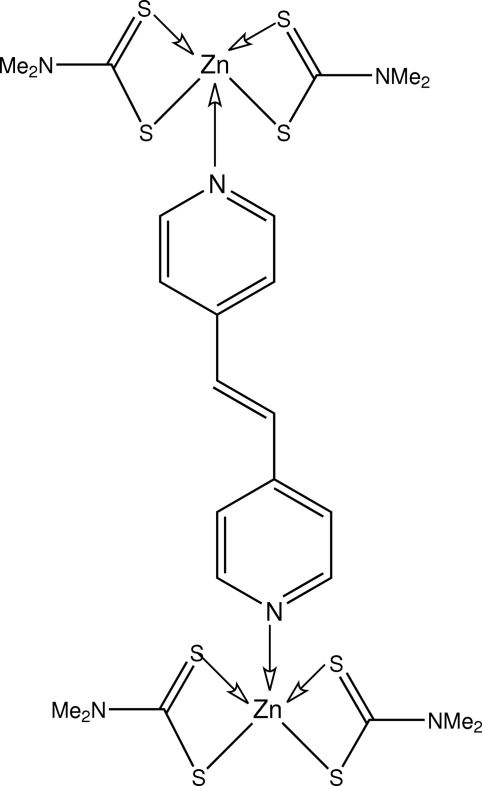


## Experimental

### 

#### Crystal data

[Zn_2_(C_3_H_6_NS_2_)_4_(C_12_H_10_N_2_)]
*M* *_r_* = 793.79
Monoclinic, 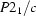

*a* = 13.061 (4) Å
*b* = 15.904 (4) Å
*c* = 17.658 (5) Å
β = 108.443 (4)°
*V* = 3479.7 (16) Å^3^
*Z* = 4
Mo *K*α radiation
μ = 1.88 mm^−1^
*T* = 98 K
0.40 × 0.08 × 0.06 mm

#### Data collection

Rigaku AFC12K/SATURN724 diffractometer
Absorption correction: multi-scan (*ABSCOR*; Higashi, 1995 ▶) *T*_min_ = 0.645, *T*_max_ = 1.000
23158 measured reflections
7142 independent reflections
6509 reflections with *I* > 2σ(*I*)
*R*_int_ = 0.048

#### Refinement

*R*[*F* ^2^ > 2σ(*F* ^2^)] = 0.062
*wR*(*F* ^2^) = 0.155
*S* = 1.22
7142 reflections
369 parameters
H-atom parameters constrained
Δρ_max_ = 1.51 e Å^−3^
Δρ_min_ = −0.73 e Å^−3^

### 

Data collection: *CrystalClear* (Rigaku/MSC, 2005 ▶); cell refinement: *CrystalClear*; data reduction: *CrystalClear*; program(s) used to solve structure: *SHELXS97* (Sheldrick, 2008 ▶); program(s) used to refine structure: *SHELXL97* (Sheldrick, 2008 ▶); molecular graphics: *ORTEPII* (Johnson, 1976 ▶) and *DIAMOND* (Brandenburg, 2006 ▶); software used to prepare material for publication: *SHELXL97*.

## Supplementary Material

Crystal structure: contains datablocks global, I. DOI: 10.1107/S1600536809044249/pv2225sup1.cif

Structure factors: contains datablocks I. DOI: 10.1107/S1600536809044249/pv2225Isup2.hkl

Additional supplementary materials:  crystallographic information; 3D view; checkCIF report

## Figures and Tables

**Table 1:** Hydrogen-bond geometry (Å, °)

*D*—H⋯*A*	*D*—H	H⋯*A*	*D*⋯*A*	*D*—H⋯*A*
C13—H13⋯S6^i^	0.95	2.81	3.636 (5)	146
C18—H18⋯*Cg*1^ii^	0.95	2.76	3.589 (5)	146
C24—H24b⋯*Cg*2^iii^	0.98	2.93	3.638 (7)	130

Symmetry codes: (i) 
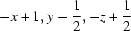
; (ii) 
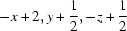
; (iii) 
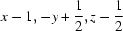
. *Cg*1 is the centroid of the Zn, S1, S2, C1 chelate ring and *Cg*2 is the centroid of the N3, C7–C11 ring.

**Fig. 1.**
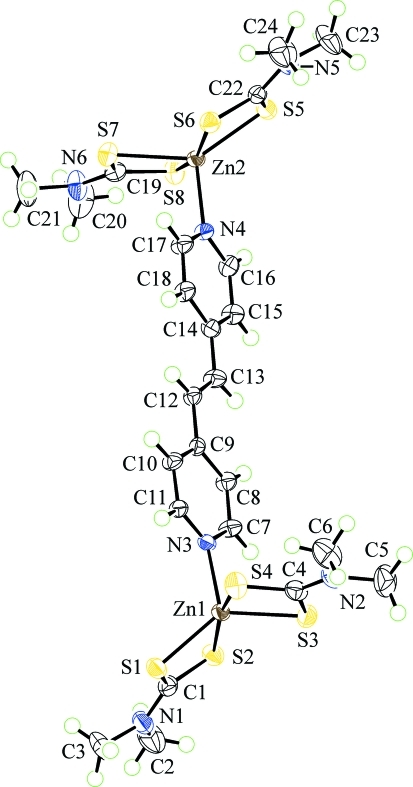
Molecular structure of (I) showing the crystallographic numbering scheme. Displacement ellipsoids are shown at the 70% probability level.

**Fig. 2.**
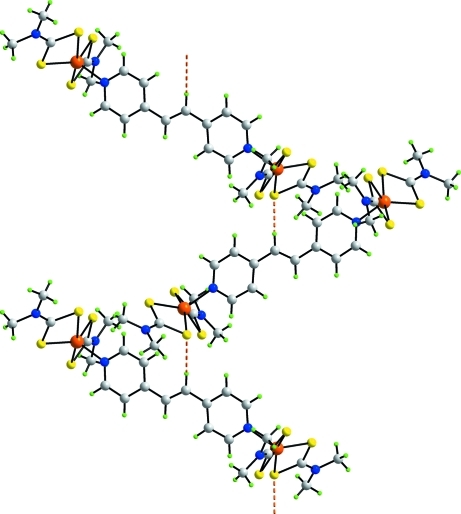
Supramolecular chain in (I) mediated by C—H···S interactions (orange dashed lines).

**Fig. 3.**
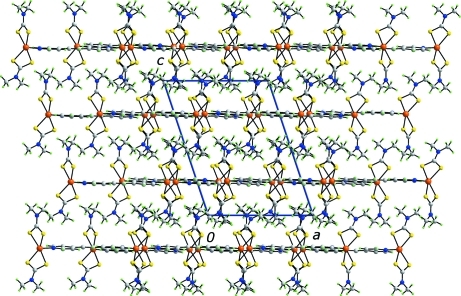
Unit-cell contents for (I) viewed in projection down the b axis.

## supplementary crystallographic information

### Comment

Compared to their xanthates (^-^S_2_COR) and dithiophosphates [^-^S_2_P(OR)_2_], crystal engineering studies of zinc(II) dithiocarbamates (^-^S_2_CN*R*_2_) are less well developed (Lai *et al.*, 2002; Chen *et al.*, 2006; Benson *et al.* 2007). This is likely due to the stronger chelating ability of the dithiocarbamate ligand which tends to preclude incorporation of multiple bridging ligands within the Zn atom coordination sphere. This principle is exemplified in the title compound, (I), Fig. 1, where each Zn atom is five coordinate within a NS_4_ donor set. The dithiocarbamate ligands are chelating but form disparate Zn—S bond distances ranging from 2.3204 (15) to 2.6650 (16) Å. The coordination geometries for the Zn1 and Zn2 atoms are distorted towards trigonal bipyramidal (TP) and square pyramidal (SP), respectively. This is quantified by the values of τ = 0.58 and 0.39, respectively, compared with the ideal values of 0.0 and 1.0 for SP and TP, respectively (Addison *et al.*, 1984).

The most closely related structure available for comparison is the diethyldithiocarbamate analogue of (I) which was co-crystallized with a *trans*-1,2-bis(4-pyridyl)ethylene molecule (Lai & Tiekink, 2003). Here, the range of Zn—S bond distances was considerably narrower, *i.e.* 2.4100 (10) to 2.4914 (11) Å, and the coordination geometry was close to SP (τ = 0.13).

Molecules of (I) are connected by C—H···S interactions, Table 1, to form supramolecular zigzag chains that pack in the *ab* plane, Table 1 and Fig. 2. Chains are connected *via* C—H···π interactions to consolidate the crystal packing, Table 1 and Fig. 3.

### Experimental

Compound (I) was prepared by following a standard literature procedure (Lai & Tiekink, 2003). and recrystallized from the slow evaporation of a chloroform/acetonitrile (3:1) solution of (I); m. pt. 555–557 K.

### Refinement

The H atoms were geometrically placed (C—H = 0.95–0.98 Å) and refined as riding with *U*_iso_(H) = 1.2–1.5*U*_eq_(C). The maximum and minimum residual electron density peaks of 1.51 and 0.73 e Å^-3^, respectively, were located 1.81 Å and 1.17 Å from the S5 and Zn2 atoms, respectively.

### Crystal data

**Table:**

[Zn_2_(C_3_H_6_NS_2_)_4_(C_12_H_10_N_2_)]	*F*(000) = 1632
*M**_r_* = 793.79	*D*_x_ = 1.515 Mg m^−^^3^
Monoclinic, *P*2_1_/*c*	Mo *K*α radiation, λ = 0.71069 Å
Hall symbol: -P 2ybc	Cell parameters from 17793 reflections
*a* = 13.061 (4) Å	θ = 2.1–40.7°
*b* = 15.904 (4) Å	µ = 1.88 mm^−^^1^
*c* = 17.658 (5) Å	*T* = 98 K
β = 108.443 (4)°	Prism, pale-yellow
*V* = 3479.7 (16) Å^3^	0.40 × 0.08 × 0.06 mm
*Z* = 4	

### Data collection

**Table:**

Rigaku AFC12K/SATURN724 diffractometer	7142 independent reflections
Radiation source: fine-focus sealed tube	6509 reflections with *I* > 2σ(*I*)
graphite	*R*_int_ = 0.048
ω scans	θ_max_ = 26.5°, θ_min_ = 2.1°
Absorption correction: multi-scan (*ABSCOR*; Higashi, 1995)	*h* = −14→16
*T*_min_ = 0.645, *T*_max_ = 1	*k* = −19→19
23158 measured reflections	*l* = −22→22

### Refinement

**Table:**

Refinement on *F*^2^	Primary atom site location: structure-invariant direct methods
Least-squares matrix: full	Secondary atom site location: difference Fourier map
*R*[*F*^2^ > 2σ(*F*^2^)] = 0.062	Hydrogen site location: inferred from neighbouring sites
*wR*(*F*^2^) = 0.155	H-atom parameters constrained
*S* = 1.22	*w* = 1/[σ^2^(*F*_o_^2^) + (0.0348*P*)^2^ + 22.73*P*] where *P* = (*F*_o_^2^ + 2*F*_c_^2^)/3
7142 reflections	(Δ/σ)_max_ = 0.001
369 parameters	Δρ_max_ = 1.51 e Å^−^^3^
0 restraints	Δρ_min_ = −0.73 e Å^−^^3^

### Special details

**Table:**

Geometry. All e.s.d.'s (except the e.s.d. in the dihedral angle between two l.s. planes) are estimated using the full covariance matrix. The cell e.s.d.'s are taken into account individually in the estimation of e.s.d.'s in distances, angles and torsion angles; correlations between e.s.d.'s in cell parameters are only used when they are defined by crystal symmetry. An approximate (isotropic) treatment of cell e.s.d.'s is used for estimating e.s.d.'s involving l.s. planes.
Refinement. Refinement of *F*^2^ against ALL reflections. The weighted *R*-factor *wR* and goodness of fit *S* are based on *F*^2^, conventional *R*-factors *R* are based on *F*, with *F* set to zero for negative *F*^2^. The threshold expression of *F*^2^ > σ(*F*^2^) is used only for calculating *R*-factors(gt) *etc*. and is not relevant to the choice of reflections for refinement. *R*-factors based on *F*^2^ are statistically about twice as large as those based on *F*, and *R*- factors based on ALL data will be even larger.

### Fractional atomic coordinates and isotropic or equivalent isotropic displacement parameters (Å^2^)

**Table:**

	*x*	*y*	*z*	*U*_iso_*/*U*_eq_	
Zn1	1.20505 (5)	−0.06078 (4)	0.25194 (4)	0.01739 (16)	
Zn2	0.28728 (5)	0.32085 (4)	0.25404 (3)	0.01633 (15)	
S1	1.36946 (11)	−0.05320 (9)	0.35297 (8)	0.0220 (3)	
S2	1.19681 (11)	−0.16805 (9)	0.36581 (8)	0.0231 (3)	
S3	1.13226 (12)	−0.14634 (9)	0.13829 (8)	0.0249 (3)	
S4	1.23185 (12)	0.02060 (10)	0.13722 (9)	0.0270 (3)	
S5	0.12699 (11)	0.30053 (9)	0.14792 (8)	0.0217 (3)	
S6	0.29923 (11)	0.41474 (9)	0.13505 (8)	0.0219 (3)	
S7	0.36844 (12)	0.41455 (8)	0.36276 (8)	0.0212 (3)	
S8	0.24259 (12)	0.26126 (9)	0.37027 (8)	0.0235 (3)	
N1	1.3939 (4)	−0.1618 (3)	0.4724 (3)	0.0263 (11)	
N2	1.1227 (4)	−0.0632 (4)	0.0052 (3)	0.0313 (12)	
N3	1.0784 (3)	0.0114 (3)	0.2611 (2)	0.0162 (9)	
N4	0.4200 (3)	0.2477 (3)	0.2579 (3)	0.0166 (9)	
N5	0.1104 (4)	0.3966 (3)	0.0203 (3)	0.0297 (11)	
N6	0.3653 (4)	0.3445 (3)	0.4985 (3)	0.0268 (11)	
C1	1.3264 (5)	−0.1315 (3)	0.4054 (3)	0.0215 (11)	
C2	1.3594 (6)	−0.2265 (5)	0.5182 (4)	0.0422 (17)	
H2A	1.3048	−0.2031	0.5395	0.063*	
H2B	1.4217	−0.2456	0.5623	0.063*	
H2C	1.3287	−0.2743	0.4832	0.063*	
C3	1.5023 (5)	−0.1256 (5)	0.5090 (4)	0.0394 (16)	
H3A	1.5345	−0.1125	0.4672	0.059*	
H3B	1.5480	−0.1663	0.5464	0.059*	
H3C	1.4966	−0.0740	0.5377	0.059*	
C4	1.1581 (5)	−0.0622 (4)	0.0845 (3)	0.0237 (12)	
C5	1.0612 (7)	−0.1339 (5)	−0.0405 (4)	0.0448 (18)	
H5A	1.1110	−0.1749	−0.0511	0.067*	
H5B	1.0118	−0.1132	−0.0912	0.067*	
H5C	1.0196	−0.1608	−0.0097	0.067*	
C6	1.1407 (6)	0.0060 (5)	−0.0437 (4)	0.0405 (17)	
H6A	1.1687	0.0549	−0.0096	0.061*	
H6B	1.0723	0.0209	−0.0842	0.061*	
H6C	1.1930	−0.0113	−0.0700	0.061*	
C7	0.9896 (4)	−0.0291 (3)	0.2653 (3)	0.0205 (11)	
H7	0.9906	−0.0888	0.2679	0.025*	
C8	0.8978 (4)	0.0128 (3)	0.2659 (3)	0.0195 (11)	
H8	0.8369	−0.0181	0.2689	0.023*	
C9	0.8936 (4)	0.1001 (3)	0.2622 (3)	0.0155 (10)	
C10	0.9858 (4)	0.1419 (3)	0.2589 (3)	0.0187 (10)	
H10	0.9873	0.2015	0.2576	0.022*	
C11	1.0749 (4)	0.0960 (3)	0.2577 (3)	0.0172 (10)	
H11	1.1366	0.1255	0.2544	0.021*	
C12	0.7970 (4)	0.1474 (3)	0.2610 (3)	0.0171 (10)	
H12	0.8001	0.2070	0.2581	0.020*	
C13	0.7048 (4)	0.1132 (3)	0.2637 (3)	0.0203 (11)	
H13	0.7021	0.0536	0.2672	0.024*	
C14	0.6081 (4)	0.1601 (3)	0.2616 (3)	0.0188 (11)	
C15	0.5180 (4)	0.1187 (3)	0.2703 (3)	0.0206 (11)	
H15	0.5195	0.0595	0.2777	0.025*	
C16	0.4266 (4)	0.1642 (3)	0.2681 (3)	0.0200 (11)	
H16	0.3661	0.1350	0.2741	0.024*	
C17	0.5068 (4)	0.2883 (3)	0.2503 (4)	0.0221 (11)	
H17	0.5032	0.3477	0.2437	0.027*	
C18	0.6004 (4)	0.2477 (3)	0.2516 (3)	0.0202 (11)	
H18	0.6595	0.2788	0.2457	0.024*	
C19	0.3290 (4)	0.3405 (3)	0.4199 (3)	0.0202 (11)	
C20	0.3321 (7)	0.2847 (4)	0.5497 (4)	0.0415 (18)	
H20A	0.2795	0.2451	0.5163	0.062*	
H20B	0.2993	0.3154	0.5843	0.062*	
H20C	0.3954	0.2537	0.5827	0.062*	
C21	0.4428 (6)	0.4077 (4)	0.5421 (4)	0.0378 (16)	
H21A	0.4786	0.4328	0.5064	0.057*	
H21B	0.4969	0.3811	0.5874	0.057*	
H21C	0.4049	0.4516	0.5617	0.057*	
C22	0.1729 (4)	0.3733 (3)	0.0934 (3)	0.0198 (11)	
C23	0.0051 (6)	0.3582 (5)	−0.0177 (4)	0.0414 (17)	
H23A	0.0144	0.3056	−0.0438	0.062*	
H23B	−0.0401	0.3969	−0.0577	0.062*	
H23C	−0.0298	0.3463	0.0228	0.062*	
C24	0.1514 (6)	0.4526 (5)	−0.0292 (4)	0.0407 (17)	
H24A	0.1989	0.4948	0.0047	0.061*	
H24B	0.0907	0.4807	−0.0685	0.061*	
H24C	0.1920	0.4197	−0.0571	0.061*	

### Atomic displacement parameters (Å^2^)

**Table:**

	*U*^11^	*U*^22^	*U*^33^	*U*^12^	*U*^13^	*U*^23^
Zn1	0.0141 (3)	0.0210 (3)	0.0169 (3)	0.0026 (2)	0.0046 (2)	−0.0017 (2)
Zn2	0.0149 (3)	0.0178 (3)	0.0163 (3)	0.0025 (2)	0.0051 (2)	−0.0006 (2)
S1	0.0173 (6)	0.0239 (7)	0.0221 (7)	−0.0011 (5)	0.0025 (5)	−0.0001 (5)
S2	0.0225 (7)	0.0234 (7)	0.0220 (7)	−0.0002 (5)	0.0050 (5)	0.0024 (5)
S3	0.0322 (8)	0.0199 (7)	0.0203 (7)	0.0009 (6)	0.0049 (6)	−0.0016 (5)
S4	0.0298 (8)	0.0309 (8)	0.0222 (7)	−0.0065 (6)	0.0110 (6)	−0.0001 (6)
S5	0.0188 (7)	0.0251 (7)	0.0195 (7)	0.0007 (5)	0.0038 (5)	−0.0004 (5)
S6	0.0241 (7)	0.0219 (7)	0.0198 (7)	0.0013 (5)	0.0071 (5)	−0.0004 (5)
S7	0.0290 (7)	0.0187 (6)	0.0175 (6)	−0.0041 (5)	0.0097 (5)	0.0004 (5)
S8	0.0244 (7)	0.0258 (7)	0.0224 (7)	−0.0087 (6)	0.0102 (6)	−0.0022 (5)
N1	0.025 (3)	0.027 (3)	0.022 (2)	−0.001 (2)	0.000 (2)	0.000 (2)
N2	0.034 (3)	0.038 (3)	0.019 (2)	0.007 (2)	0.005 (2)	0.002 (2)
N3	0.013 (2)	0.019 (2)	0.015 (2)	0.0025 (17)	0.0028 (16)	0.0002 (17)
N4	0.017 (2)	0.014 (2)	0.019 (2)	0.0017 (17)	0.0058 (17)	0.0012 (17)
N5	0.032 (3)	0.034 (3)	0.019 (2)	−0.001 (2)	0.002 (2)	0.002 (2)
N6	0.039 (3)	0.027 (3)	0.014 (2)	−0.012 (2)	0.008 (2)	0.0019 (19)
C1	0.026 (3)	0.021 (3)	0.017 (3)	0.002 (2)	0.006 (2)	−0.001 (2)
C2	0.047 (4)	0.045 (4)	0.025 (3)	−0.005 (3)	−0.001 (3)	0.012 (3)
C3	0.029 (3)	0.047 (4)	0.031 (3)	−0.008 (3)	−0.008 (3)	0.004 (3)
C4	0.022 (3)	0.031 (3)	0.017 (3)	0.006 (2)	0.006 (2)	0.001 (2)
C5	0.060 (5)	0.046 (4)	0.021 (3)	0.000 (4)	0.003 (3)	−0.007 (3)
C6	0.043 (4)	0.056 (5)	0.022 (3)	0.000 (3)	0.010 (3)	0.008 (3)
C7	0.019 (3)	0.018 (3)	0.027 (3)	0.004 (2)	0.011 (2)	0.003 (2)
C8	0.018 (3)	0.015 (2)	0.026 (3)	−0.002 (2)	0.008 (2)	0.001 (2)
C9	0.019 (3)	0.014 (2)	0.013 (2)	−0.0002 (19)	0.0041 (19)	−0.0012 (18)
C10	0.019 (3)	0.013 (2)	0.025 (3)	0.001 (2)	0.007 (2)	0.002 (2)
C11	0.014 (2)	0.014 (2)	0.023 (3)	−0.0008 (19)	0.006 (2)	0.000 (2)
C12	0.017 (3)	0.014 (2)	0.021 (3)	−0.0019 (19)	0.006 (2)	−0.0005 (19)
C13	0.020 (3)	0.014 (2)	0.027 (3)	0.000 (2)	0.008 (2)	0.006 (2)
C14	0.017 (3)	0.018 (3)	0.020 (3)	0.000 (2)	0.004 (2)	0.002 (2)
C15	0.021 (3)	0.017 (3)	0.025 (3)	−0.002 (2)	0.009 (2)	0.005 (2)
C16	0.022 (3)	0.017 (3)	0.022 (3)	−0.003 (2)	0.009 (2)	0.001 (2)
C17	0.018 (3)	0.013 (2)	0.036 (3)	−0.002 (2)	0.010 (2)	0.000 (2)
C18	0.020 (3)	0.014 (2)	0.028 (3)	−0.003 (2)	0.010 (2)	0.002 (2)
C19	0.021 (3)	0.020 (3)	0.022 (3)	0.001 (2)	0.009 (2)	0.001 (2)
C20	0.072 (5)	0.031 (3)	0.026 (3)	−0.019 (3)	0.022 (3)	0.005 (3)
C21	0.052 (4)	0.040 (4)	0.020 (3)	−0.018 (3)	0.010 (3)	−0.005 (3)
C22	0.021 (3)	0.021 (3)	0.016 (3)	0.005 (2)	0.004 (2)	−0.002 (2)
C23	0.037 (4)	0.053 (4)	0.022 (3)	−0.002 (3)	−0.007 (3)	0.008 (3)
C24	0.048 (4)	0.045 (4)	0.025 (3)	−0.001 (3)	0.005 (3)	0.011 (3)

### Geometric parameters (Å, °)

**Table:**

Zn1—N3	2.061 (4)	C5—H5A	0.9800
Zn1—S1	2.3204 (15)	C5—H5B	0.9800
Zn1—S3	2.3613 (16)	C5—H5C	0.9800
Zn1—S4	2.5200 (16)	C6—H6A	0.9800
Zn1—S2	2.6650 (16)	C6—H6B	0.9800
Zn2—N4	2.071 (4)	C6—H6C	0.9800
Zn2—S5	2.3488 (15)	C7—C8	1.376 (7)
Zn2—S7	2.3964 (15)	C7—H7	0.9500
Zn2—S8	2.4918 (16)	C8—C9	1.391 (7)
Zn2—S6	2.6223 (16)	C8—H8	0.9500
S1—C1	1.746 (6)	C9—C10	1.391 (7)
S2—C1	1.716 (6)	C9—C12	1.463 (7)
S3—C4	1.736 (6)	C10—C11	1.380 (7)
S4—C4	1.721 (6)	C10—H10	0.9500
S5—C22	1.730 (6)	C11—H11	0.9500
S6—C22	1.711 (6)	C12—C13	1.336 (7)
S7—C19	1.732 (6)	C12—H12	0.9500
S8—C19	1.733 (6)	C13—C14	1.458 (7)
N1—C1	1.323 (7)	C13—H13	0.9500
N1—C2	1.466 (8)	C14—C15	1.399 (7)
N1—C3	1.475 (8)	C14—C18	1.403 (7)
N2—C4	1.328 (7)	C15—C16	1.387 (8)
N2—C6	1.463 (9)	C15—H15	0.9500
N2—C5	1.466 (9)	C16—H16	0.9500
N3—C11	1.348 (7)	C17—C18	1.377 (8)
N3—C7	1.348 (7)	C17—H17	0.9500
N4—C16	1.339 (7)	C18—H18	0.9500
N4—C17	1.349 (7)	C20—H20A	0.9800
N5—C22	1.343 (7)	C20—H20B	0.9800
N5—C23	1.460 (8)	C20—H20C	0.9800
N5—C24	1.463 (8)	C21—H21A	0.9800
N6—C19	1.319 (7)	C21—H21B	0.9800
N6—C21	1.460 (8)	C21—H21C	0.9800
N6—C20	1.470 (7)	C23—H23A	0.9800
C2—H2A	0.9800	C23—H23B	0.9800
C2—H2B	0.9800	C23—H23C	0.9800
C2—H2C	0.9800	C24—H24A	0.9800
C3—H3A	0.9800	C24—H24B	0.9800
C3—H3B	0.9800	C24—H24C	0.9800
C3—H3C	0.9800		
			
N3—Zn1—S1	118.77 (13)	N2—C6—H6B	109.5
N3—Zn1—S3	105.66 (13)	H6A—C6—H6B	109.5
S1—Zn1—S3	135.17 (6)	N2—C6—H6C	109.5
N3—Zn1—S4	95.51 (13)	H6A—C6—H6C	109.5
S1—Zn1—S4	105.14 (6)	H6B—C6—H6C	109.5
S3—Zn1—S4	74.56 (6)	N3—C7—C8	122.5 (5)
N3—Zn1—S2	94.15 (13)	N3—C7—H7	118.8
S1—Zn1—S2	72.64 (5)	C8—C7—H7	118.8
S3—Zn1—S2	99.84 (6)	C7—C8—C9	120.3 (5)
S4—Zn1—S2	169.86 (5)	C7—C8—H8	119.9
N4—Zn2—S5	118.30 (13)	C9—C8—H8	119.9
N4—Zn2—S7	99.78 (13)	C8—C9—C10	117.2 (5)
S5—Zn2—S7	141.40 (6)	C8—C9—C12	122.3 (5)
N4—Zn2—S8	99.85 (13)	C10—C9—C12	120.5 (5)
S5—Zn2—S8	102.88 (6)	C11—C10—C9	119.6 (5)
S7—Zn2—S8	74.71 (5)	C11—C10—H10	120.2
N4—Zn2—S6	94.87 (12)	C9—C10—H10	120.2
S5—Zn2—S6	72.73 (5)	N3—C11—C10	123.0 (5)
S7—Zn2—S6	99.46 (5)	N3—C11—H11	118.5
S8—Zn2—S6	164.88 (5)	C10—C11—H11	118.5
C1—S1—Zn1	89.75 (19)	C13—C12—C9	124.9 (5)
C1—S2—Zn1	79.62 (19)	C13—C12—H12	117.5
C4—S3—Zn1	85.7 (2)	C9—C12—H12	117.5
C4—S4—Zn1	81.14 (19)	C12—C13—C14	125.0 (5)
C22—S5—Zn2	88.47 (19)	C12—C13—H13	117.5
C22—S6—Zn2	80.31 (19)	C14—C13—H13	117.5
C19—S7—Zn2	84.58 (19)	C15—C14—C18	116.9 (5)
C19—S8—Zn2	81.66 (18)	C15—C14—C13	120.5 (5)
C1—N1—C2	121.0 (5)	C18—C14—C13	122.6 (5)
C1—N1—C3	121.7 (5)	C16—C15—C14	119.8 (5)
C2—N1—C3	116.9 (5)	C16—C15—H15	120.1
C4—N2—C6	123.0 (6)	C14—C15—H15	120.1
C4—N2—C5	122.4 (6)	N4—C16—C15	122.7 (5)
C6—N2—C5	114.5 (5)	N4—C16—H16	118.7
C11—N3—C7	117.5 (5)	C15—C16—H16	118.7
C11—N3—Zn1	124.7 (4)	N4—C17—C18	122.9 (5)
C7—N3—Zn1	117.7 (4)	N4—C17—H17	118.6
C16—N4—C17	118.0 (5)	C18—C17—H17	118.6
C16—N4—Zn2	125.4 (4)	C17—C18—C14	119.8 (5)
C17—N4—Zn2	116.6 (4)	C17—C18—H18	120.1
C22—N5—C23	121.7 (5)	C14—C18—H18	120.1
C22—N5—C24	121.0 (5)	N6—C19—S7	120.3 (4)
C23—N5—C24	116.7 (5)	N6—C19—S8	121.9 (4)
C19—N6—C21	123.2 (5)	S7—C19—S8	117.8 (3)
C19—N6—C20	122.5 (5)	N6—C20—H20A	109.5
C21—N6—C20	114.3 (5)	N6—C20—H20B	109.5
N1—C1—S2	122.2 (4)	H20A—C20—H20B	109.5
N1—C1—S1	119.9 (4)	N6—C20—H20C	109.5
S2—C1—S1	117.9 (3)	H20A—C20—H20C	109.5
N1—C2—H2A	109.5	H20B—C20—H20C	109.5
N1—C2—H2B	109.5	N6—C21—H21A	109.5
H2A—C2—H2B	109.5	N6—C21—H21B	109.5
N1—C2—H2C	109.5	H21A—C21—H21B	109.5
H2A—C2—H2C	109.5	N6—C21—H21C	109.5
H2B—C2—H2C	109.5	H21A—C21—H21C	109.5
N1—C3—H3A	109.5	H21B—C21—H21C	109.5
N1—C3—H3B	109.5	N5—C22—S6	121.2 (4)
H3A—C3—H3B	109.5	N5—C22—S5	120.4 (4)
N1—C3—H3C	109.5	S6—C22—S5	118.4 (3)
H3A—C3—H3C	109.5	N5—C23—H23A	109.5
H3B—C3—H3C	109.5	N5—C23—H23B	109.5
N2—C4—S4	121.9 (5)	H23A—C23—H23B	109.5
N2—C4—S3	120.3 (5)	N5—C23—H23C	109.5
S4—C4—S3	117.8 (3)	H23A—C23—H23C	109.5
N2—C5—H5A	109.5	H23B—C23—H23C	109.5
N2—C5—H5B	109.5	N5—C24—H24A	109.5
H5A—C5—H5B	109.5	N5—C24—H24B	109.5
N2—C5—H5C	109.5	H24A—C24—H24B	109.5
H5A—C5—H5C	109.5	N5—C24—H24C	109.5
H5B—C5—H5C	109.5	H24A—C24—H24C	109.5
N2—C6—H6A	109.5	H24B—C24—H24C	109.5
			
N3—Zn1—S1—C1	87.2 (2)	Zn1—S2—C1—S1	3.1 (3)
S3—Zn1—S1—C1	−84.5 (2)	Zn1—S1—C1—N1	175.1 (5)
S4—Zn1—S1—C1	−167.63 (19)	Zn1—S1—C1—S2	−3.5 (3)
S2—Zn1—S1—C1	2.06 (18)	C6—N2—C4—S4	−1.6 (9)
N3—Zn1—S2—C1	−121.0 (2)	C5—N2—C4—S4	179.1 (5)
S1—Zn1—S2—C1	−2.13 (19)	C6—N2—C4—S3	179.3 (5)
S3—Zn1—S2—C1	132.29 (19)	C5—N2—C4—S3	−0.1 (9)
S4—Zn1—S2—C1	76.7 (4)	Zn1—S4—C4—N2	172.6 (5)
N3—Zn1—S3—C4	86.0 (2)	Zn1—S4—C4—S3	−8.2 (3)
S1—Zn1—S3—C4	−101.6 (2)	Zn1—S3—C4—N2	−172.1 (5)
S4—Zn1—S3—C4	−5.44 (19)	Zn1—S3—C4—S4	8.7 (3)
S2—Zn1—S3—C4	−176.78 (19)	C11—N3—C7—C8	0.2 (8)
N3—Zn1—S4—C4	−99.2 (2)	Zn1—N3—C7—C8	−175.4 (4)
S1—Zn1—S4—C4	139.0 (2)	N3—C7—C8—C9	0.0 (8)
S3—Zn1—S4—C4	5.5 (2)	C7—C8—C9—C10	−0.8 (8)
S2—Zn1—S4—C4	63.0 (4)	C7—C8—C9—C12	178.7 (5)
N4—Zn2—S5—C22	−88.2 (2)	C8—C9—C10—C11	1.4 (8)
S7—Zn2—S5—C22	81.4 (2)	C12—C9—C10—C11	−178.1 (5)
S8—Zn2—S5—C22	162.95 (18)	C7—N3—C11—C10	0.4 (8)
S6—Zn2—S5—C22	−2.06 (18)	Zn1—N3—C11—C10	175.7 (4)
N4—Zn2—S6—C22	120.3 (2)	C9—C10—C11—N3	−1.3 (8)
S5—Zn2—S6—C22	2.11 (18)	C8—C9—C12—C13	0.4 (8)
S7—Zn2—S6—C22	−138.96 (18)	C10—C9—C12—C13	179.8 (5)
S8—Zn2—S6—C22	−73.1 (3)	C9—C12—C13—C14	−179.3 (5)
N4—Zn2—S7—C19	−90.9 (2)	C12—C13—C14—C15	−175.5 (5)
S5—Zn2—S7—C19	98.4 (2)	C12—C13—C14—C18	4.0 (9)
S8—Zn2—S7—C19	6.76 (19)	C18—C14—C15—C16	0.6 (8)
S6—Zn2—S7—C19	172.47 (19)	C13—C14—C15—C16	−179.9 (5)
N4—Zn2—S8—C19	90.7 (2)	C17—N4—C16—C15	−0.7 (8)
S5—Zn2—S8—C19	−147.03 (19)	Zn2—N4—C16—C15	−179.8 (4)
S7—Zn2—S8—C19	−6.80 (19)	C14—C15—C16—N4	0.0 (8)
S6—Zn2—S8—C19	−75.8 (3)	C16—N4—C17—C18	0.9 (8)
S1—Zn1—N3—C11	66.9 (4)	Zn2—N4—C17—C18	−179.9 (4)
S3—Zn1—N3—C11	−119.2 (4)	N4—C17—C18—C14	−0.4 (9)
S4—Zn1—N3—C11	−43.7 (4)	C15—C14—C18—C17	−0.4 (8)
S2—Zn1—N3—C11	139.4 (4)	C13—C14—C18—C17	−179.9 (5)
S1—Zn1—N3—C7	−117.8 (4)	C21—N6—C19—S7	−2.6 (9)
S3—Zn1—N3—C7	56.1 (4)	C20—N6—C19—S7	178.3 (5)
S4—Zn1—N3—C7	131.6 (4)	C21—N6—C19—S8	176.5 (5)
S2—Zn1—N3—C7	−45.3 (4)	C20—N6—C19—S8	−2.6 (9)
S5—Zn2—N4—C16	−65.3 (5)	Zn2—S7—C19—N6	168.5 (5)
S7—Zn2—N4—C16	121.2 (4)	Zn2—S7—C19—S8	−10.6 (3)
S8—Zn2—N4—C16	45.2 (4)	Zn2—S8—C19—N6	−168.9 (5)
S6—Zn2—N4—C16	−138.3 (4)	Zn2—S8—C19—S7	10.3 (3)
S5—Zn2—N4—C17	115.6 (4)	C23—N5—C22—S6	176.4 (5)
S7—Zn2—N4—C17	−57.9 (4)	C24—N5—C22—S6	5.4 (8)
S8—Zn2—N4—C17	−133.9 (4)	C23—N5—C22—S5	−3.7 (8)
S6—Zn2—N4—C17	42.6 (4)	C24—N5—C22—S5	−174.8 (5)
C2—N1—C1—S2	−2.5 (8)	Zn2—S6—C22—N5	176.8 (5)
C3—N1—C1—S2	−175.3 (5)	Zn2—S6—C22—S5	−3.1 (3)
C2—N1—C1—S1	179.0 (5)	Zn2—S5—C22—N5	−176.4 (5)
C3—N1—C1—S1	6.2 (8)	Zn2—S5—C22—S6	3.4 (3)
Zn1—S2—C1—N1	−175.5 (5)		

### Hydrogen-bond geometry (Å, °)

**Table:**

*D*—H···*A*	*D*—H	H···*A*	*D*···*A*	*D*—H···*A*
C13—H13···S6^i^	0.95	2.81	3.636 (5)	146
C18—H18···Cg1^ii^	0.95	2.76	3.589 (5)	146
C24—H24b···Cg2^iii^	0.98	2.93	3.638 (7)	130

Symmetry codes: (i) −*x*+1, *y*−1/2, −*z*+1/2; (ii) −*x*+2, *y*+1/2, −*z*+1/2; (iii) *x*−1, −*y*+1/2, *z*−1/2.

## References

[bb1] Addison, A. W., Rao, T. N., Reedijk, J., van Rijn, J. & Verschoor, G. C. (1984). *J. Chem. Soc. Dalton Trans.* pp. 1349–1356.

[bb2] Benson, R. E., Ellis, C. A., Lewis, C. E. & Tiekink, E. R. T. (2007). *CrystEngComm*, **9**, 930–940.

[bb3] Brandenburg, K. (2006). *DIAMOND* Crystal Impact GbR, Bonn, Germany.

[bb4] Chen, D., Lai, C. S. & Tiekink, E. R. T. (2006). *CrystEngComm*, **8**, 51–58.

[bb5] Higashi, T. (1995). *ABSCOR* Rigaku Corporation, Tokyo, Japan.

[bb6] Johnson, C. K. (1976). *ORTEPII* Report ORNL-5138. Oak Ridge National Laboratory, Tennessee, USA.

[bb7] Lai, C. S., Lim, Y. X., Yap, T. C. & Tiekink, E. R. T. (2002). *CrystEngComm*, **4**, 596–600.

[bb8] Lai, C. S. & Tiekink, E. R. T. (2003). *Appl. Organomet. Chem.* **17**, 251–252.

[bb9] Rigaku/MSC (2005). *CrystalClear* Rigaku/MSC Inc., The Woodlands, Texas, USA.

[bb10] Sheldrick, G. M. (2008). *Acta Cryst.* A**64**, 112–122. 10.1107/S0108767307043930 18156677

